# Thionin-like peptide from *Capsicum annuum* fruits: mechanism of action and synergism with fluconazole against *Candida* species

**DOI:** 10.1186/s12866-016-0626-6

**Published:** 2016-01-27

**Authors:** Gabriel B. Taveira, André O. Carvalho, Rosana Rodrigues, Fernanda G. Trindade, Maura Da Cunha, Valdirene M. Gomes

**Affiliations:** Laboratório de Fisiologia e Bioquímica de Microrganismos, Centro de Biociências e Biotecnologia, Universidade Estadual do Norte Fluminense, Campos dos Goytacazes, 28013-602, RJ Brazil; Laboratório de Melhoramento Genético Vegetal, Centro de Ciências e Tecnologias Agropecuárias, Universidade Estadual do Norte Fluminense, Campos dos Goytacazes, 28013-602, RJ Brazil; Laboratório de Biologia Celular e Tecidual, Centro de Biociências e Biotecnologia, Universidade Estadual do Norte Fluminense, Campos dos Goytacazes, 28013-602, RJ Brazil

**Keywords:** Antimicrobial peptides, Thionin, Synergistic activity, Fluconazole, *Candida*

## Abstract

**Background:**

Thionins are a family of plant antimicrobial peptides (AMPs), which participate in plant defense system against pathogens. Here we describe some aspects of the *Ca*Thi thionin-like action mechanism, previously isolated from *Capsicum annuum* fruits. Thionin-like peptide was submitted to antimicrobial activity assays against *Candida* species for IC_50_ determination and synergism with fluconazole evaluation. Viability and plasma membrane permeabilization assays, induction of intracellular ROS production analysis and *Ca*Thi localization in yeast cells were also investigated.

**Results:**

*Ca*Thi had strong antimicrobial activity against six tested pathogenic *Candida* species, with IC_50_ ranging from 10 to 40 μg.mL^−1^. *Ca*Thi antimicrobial activity on *Candida* species was candidacidal. Moreover, *Ca*Thi caused plasma membrane permeabilization in all yeasts tested and induces oxidative stresses only in *Candida tropicalis. Ca*Thi was intracellularly localized in *C. albicans* and C. *tropicalis*, however localized in nuclei in *C. tropicalis*, suggesting a possible nuclear target. *Ca*Thi performed synergistically with fluconazole inhibiting all tested yeasts, reaching 100 % inhibition in *C. parapsilosis*. The inhibiting concentrations for the synergic pair ranged from 1.3 to 4.0 times below *Ca*Thi IC_50_ and from zero to 2.0 times below fluconazole IC_50._

**Conclusion:**

The results reported herein may ultimately contribute to future efforts aiming to employ this plant-derived AMP as a new therapeutic substance against yeasts.

## Background

Currently, a significant global public health threat is the emergence of pathogenic bacteria, fungi, and yeasts that are resistant to multiple antimicrobial agents. Indeed, few or no effective chemotherapies are available for infections caused by some of these resistant microorganisms [1, 2].

Alternatives to chemotherapies include antimicrobial peptides (AMPs), small molecules produced by all living organisms, which have gained considerable attention because of their potent antimicrobial activity against a broad range of microbes, including viruses, bacteria, protozoa, and fungi [3, 4]. Moreover, some kill microorganisms rapidly, are able to synergize with other AMPs and clinical antibiotics, have low toxicity to mammalian cells, and exert their microbial inhibitory activity at low concentrations. These molecules have multiple targets in the plasma membrane and also in intracellular components, which is thought to make an increase in microbial resistance more difficult [2, 5].

Promising AMPs include plant-derived thionins, a family of basic, low molecular weight (~5 kDa), cysteine-rich peptides. Various family members have high sequence similarity and structure [6–8]. Many of them are toxic against yeasts, pathogenic fungi, Gram-positive and Gram-negative bacteria, protozoa, and insects [7–10]. Like other AMPS, thionins’ antimicrobial activity relies on their interaction with phospholipids to cause membrane instability [10].

Infections caused by *Candida* species have increased substantially over the last 30 years due to the rise of AIDS, ageing population, numbers of immunocompromised patients and the extensive use of indwelling prosthetic devices [1, 11]. *Candida albicans* is the main cause of candidiasis, however, other *Candida* species such as *C. tropicalis*, *C. parapsilosis*, and *C. glabrata* are now frequently identified as human pathogens [11–13]. Antifungals, especially fluconazole (FLC), have been used with some success for the treatment of *Candida* infections; however, there are numerous reports on the emergence of strains resistant to azoles that overexpress multidrug efflux transporters [14, 15].

In a previous report [8], our research team isolated a plant-derived thionin, named *Ca*Thi, with strong antimicrobial activity against two pathogenic *Candida* species, as well as *Escherichia coli* and *Pseudomonas aeruginosa*. FLC in combination with AMPs resulting in promising therapeutic results against important human pathogens, such as *C. albicans* and *Cryptococcus neoformans*, has been demonstrated [16, 17]. In this work we investigated whether the AMP *Ca*Thi could act synergistically with FLC. This synergistic strategy could result in a more efficient response against six *Candida* strains of clinical importance, avoiding the cytotoxic effects commonly exhibited by thionins against mammalian cells [10] by using low concentrations of this AMP. We were also interested in understanding the mechanism by which plant-derived thionins affect *Candida* species, which remains partially unknown [10]. These questions are addressed in the present study.

The results reported herein may ultimately contribute to future efforts aiming to develop this plant-derived AMP as a new therapeutic substance against these pathogenic *Candida* species as well as other yeast infections.

## Results

### Determination of IC_50_ for *Ca*Thi and FLC

Initially we performed growth inhibition assays of six *Candida* species using different concentrations of FLC and thionin *Ca*Thi to determine the IC_50_ of these substances. The lowest IC_50_ for FLC was found for *C. buinensis* (0.125 μg.mL^−1^) and the highest for *C. pelliculosa* (5 μg.mL^- 1^). In the case of *Ca*Thi, 10 μg.mL^−1^ was sufficient to cause 50 % inhibition of growth of *C. albicans*, *C. tropicalis, C. parapsilosis*, and *C. buinensis* but 40 μg.mL^−1^ was necessary to achieve IC_50_ for *C. pelliculosa*. Thus, higher concentrations of both FLC and *Ca*Thi were needed to affect the growth of *C. pelliculosa*. Moreover, although the antimicrobial activity of *Ca*Thi against *Candida* species is indeed relevant, our data showed it to be lower than that observed for FLC (Table 1).

**Table 1:** IC_50_^a^ (μg.mL^−1^) of fluconazole and *Ca*Thi in different species of *Candida* respectively

Yeasts	Fluconazole	*Ca*Thi
*Candida albicans* (CE022)	1.0	10.0
*Candida tropicalis* (CE017)	1.0	10.0
*Candida parapsilosis* (CE002)	0.5	10.0
*Candida pelliculosa* (3974)	5.0	40.0
*Candida buinensis* (3982)	0.125	10.0
*Candida mogii* (4674)	2.5	20.0

^a^ represents the concentration of a drug that is required for 50 % inhibition *in vitro*

### Viability assay

**Fig. 1:**
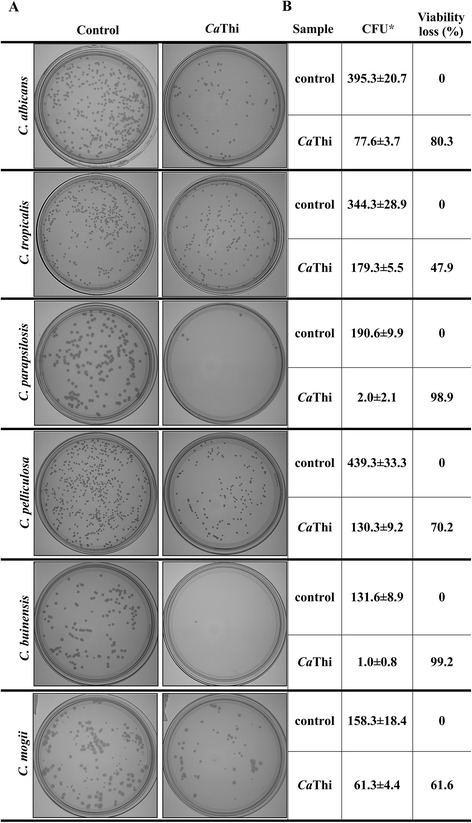
Cell viability loss. **a** Photographs of the Petri dishes showing the viability of yeasts cells after the treatment with IC_50_ *Ca*Thi for 24 h. **b** The table shows the percentage of viability loss of yeasts cells after the treatment with IC_50_ *Ca*Thi for 24 h. CFU = Colony forming unit. (*) Indicates significance by the *T* test (*P* < 0.05) among the experiments and their respective controls. The experiments were carried out in triplicate

*Ca*Thi induced viability loss in all yeasts cells tested (Fig. 1a). The most susceptible species to *Ca*Thi were *C. buinensis*, *C. parapsilosis* and *C. albicans* with 99.2, 98.9 and 80.3 % of viability loss, respectively, and the less susceptible was *C. tropicalis* with 47.9 % of viability loss (Fig. 1b). These results indicated that inhibitory effect of *Ca*Thi was candidacidal.

### Plasma membrane permeabilization

**Fig. 2:**
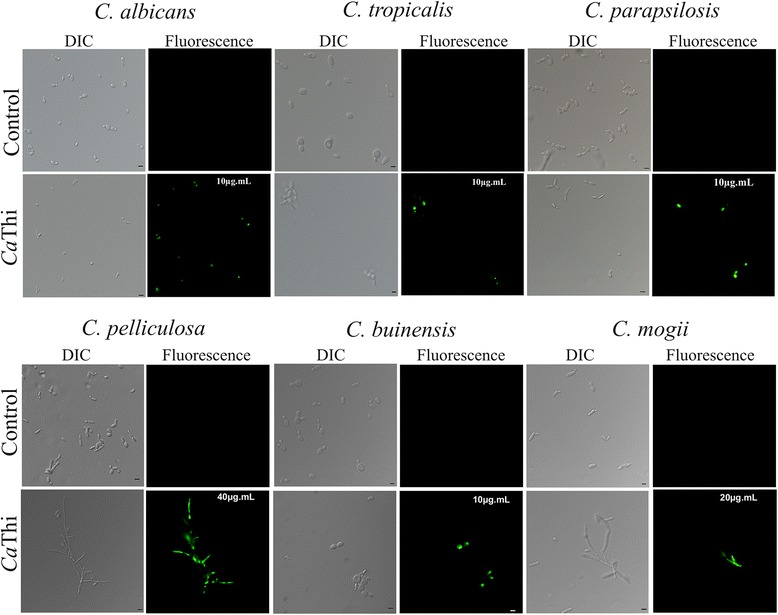
Membrane permeabilization assay. Photomicrography of different yeast cells after membrane permeabilization assay by fluorescence microscopy using the fluorescent probe Sytox green. Cells were treated with *Ca*Thi for 24 h and then assayed for membrane permeabilization. Control cells were treated only with probe Sytox green. Bars 5 μm

*Candida* species cells were tested to determine the membrane permeabilization by Sytox green dye. All yeasts showed Sytox green fluorescence when grown for 24 h in the presence of *Ca*Thi IC_50_. As with other AMPs, it is likely that *Ca*Thi acts on the plasma membrane of these *Candida* species, compromising it structurally and allowing the permeabilization of the labeling dye (Fig. 2). The membrane permeabilization percentage of the treated yeasts with *Ca*Thi was assessed (Table 2). A higher number of *C. albicans* and *C. pelliculosa* cells presented higher Sytox green fluorescence percentage, suggesting that *Ca*Thi is more effective at permeabilizing the membrane of these cells than the other *Candida* species analyzed.

**Table 2:** Fluorescent cell percentage of yeasts treated with *Ca*Thi^a^

Yeasts species	Sample	Cell number viewed in DIC	Cell number viewed in fluorescence	% of fluorescence cells^b^
*C. albicans*	control	62.0 ± 9.3	0.6 ± 0.8	0.9
	*Ca*Thi	20.0 ± 5.0	16.6 ± 5.3	83.0
*C. tropicalis*	control	41.2 ± 4.2	0.8 ± 1.3	1.9
	*Ca*Thi	9.8 ± 4.9	4.8 ± 4.6	48.9
*C. parapsilosis*	control	79.2 ± 12.1	0	0
	*Ca*Thi	18.2 ± 5.4	6.2 ± 1.4	34.0
*C. pelliculosa*	control	23.8 ± 3.5	0	0
	*Ca*Thi	7.2 ± 1.9	6.6 ± 2.3	91.6
*C. buinensis*	control	43.6 ± 9.0	0.8 ± 1.3	1.8
	*Ca*Thi	13.6 ± 6.2	6.2 ± 1.3	45.5
*C. mogii*	control	18.6 ± 3.2	0.6 ± 0.8	3.2
	*Ca*Thi	8.6 ± 2.0	4.4 ± 2.7	51.1

^a^Cells number determination in five random fields of the DIC and fluorescence views of the samples obtained from Plasma membrane permeabilization assay. The total cell number in DIC of each yeast (in control and test) was assumed as 100 %

^b^Indicates significance by the *T* test (*P* < 0.05) among the experiments and their respective controls

### ROS induction assay

**Fig. 3:**
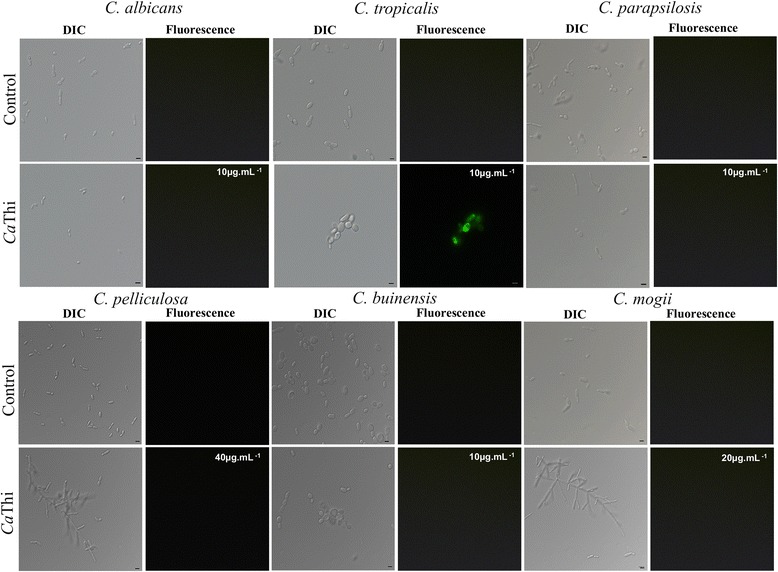
Oxidative stress assay. Photomicrography of different yeast cells after reactive oxygen species assay detection by fluorescence microscopy using the fluorescent probe 2′,7′ dichlorofluorescein diacetate (H_2_DCFDA). Cells were treated with *Ca*Thi for 24 h and then assayed for ROS detection. Control cells were treated only with probe (H_2_DCFDA). Bars 5 μm

Endogenous production of ROS was analyzed by incubating the yeasts for 24 h with *Ca*Thi IC_50_. Increased ROS production was observed only in *C. tropicalis* (Fig. 3), suggesting that a *Ca*Thi-induced increase in oxidative stress may underlie the growth inhibitory effect on this yeast. Nevertheless, oxidative stresses were not detected for other *Candida* species, implicating that we could not associate the *Ca*Thi role and ROS production with growth inhibition of *Candida*, at least for the concentrations tested.

### Localization of *Ca*Thi in yeast cells

**Fig. 4:**
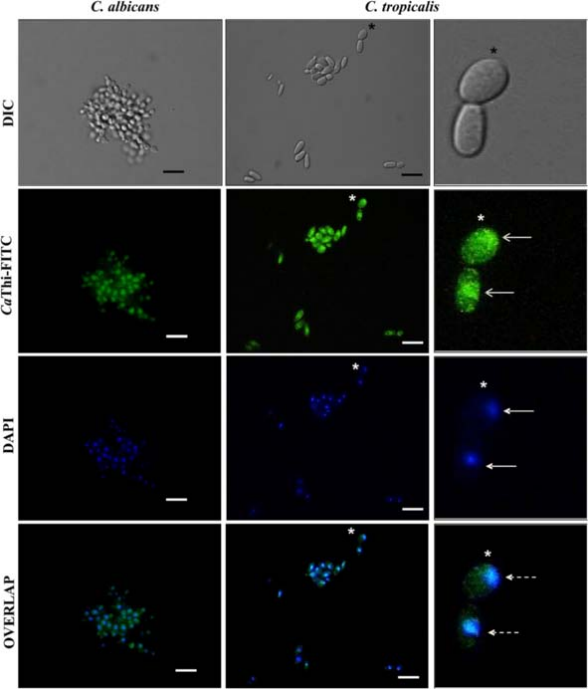
Localization of *Ca*Thi in yeast cells. Photomicrography of *Candida albicans* and *Candida tropicalis* cells incubated for 24 h with 10 μg.mL^−1^ *Ca*Thi-FITC (green fluorescence, open arrows) by fluorescence microscopy. Nuclei were visualized by 4′,6-diamidino-2-phenylindole dihydrochloride (DAPI) after the *Ca*Thi-FITC incubation period (blue fluorescence, filled arrows). Overlap of the DAPI and FITC images (dotted arrows). Bars 20 μm. (*) Indicates the position of digital enlargement

We also investigated whether *Ca*Thi was actually internalized in *C. albicans* and *C. tropicalis* cells. These yeasts were chosen because they are known to be the most opportunistic pathogens among *Candida* species. Another important point is that *C. tropicalis* was the only yeast that presented membrane permeabilization and induction of ROS by *Ca*Thi in this work. To perform the test, we used 10 μg.mL^−1^ of FITC-tagged *Ca*Thi to search for intracellular signal fluorescence. We also treated the cells with DAPI for nuclei labeling. Intracellular signal fluorescence of *Ca*Thi-FITC was observed in both of these *Candida* species. However, while *Ca*Thi-FITC labeling of *C. tropicalis* produced a specific and intense spot of fluorescence inside the cells, *C. albicans* cells showed a more diffuse fluorescence. Overlapping of these *Ca*Thi-FITC images with DAPI nuclei labeling indicated a co-localization of these fluorescent signals in *C. tropicalis* but not in *C. albicans* cells (Fig. 4). These data suggest that, at least for *C. tropicalis*, *Ca*Thi may have an intracellular target, possibly located in the nucleus.

### Synergism assay

Given the increase in *Candida* infections, particularly among immunocompromised patients, searches for antifungal therapeutic alternatives are warranted. This concern and the aforementioned data prompted us to investigate whether FLC and *Ca*Thi could act synergistically to improve therapeutic results against *Candida* species. The combination of FLC and *Ca*Thi showed an increase in inhibitory activity of all of the *Candida* species tested, suggestive of synergistic activity (Table 3). Interestingly, although *C. pelliculosa* had the highest IC_50_ for both substances, when we combined FLC at one-fold below its IC_50_ and *Ca*Thi at threefold below its IC_50,_ we observed 57 % increase in growth inhibition of this yeast. Similarly, in *C. parapsilosis* cells, when IC_50_ FLC was combined with *Ca*Thi threefold below its IC_50_, we obtained 100 % growth inhibition of this yeast. Combined use of FLC and *Ca*Thi also strongly inhibited (96 %) *C. tropicalis*, although when used separately the inhibition achieved with these substances did not reach 12 %. Taken together, these data suggest that in combination FLC and *Ca*Thi could have an important synergistic action resulting in very effective control of *Candida* species.

**Table 3:** Inhibition percentage of yeast species treated with *Ca*Thi and FLC alone and in combination showing synergism effect *in vitro*

Yeasts species	Sample	Concentration (μg.mL^−1^)^a^	Inhibition (%)	Combination inhibition (%) (*Ca*Thi + FLC)^b^
*C. albicans*	*Ca*Thi	3.5	2.93	77.5
	FLC	0.5	24.48	
*C. tropicalis*	*Ca*Thi	3.5	0	96.26
	FLC	0.5	11.55	
*C. parapsilosis*	*Ca*Thi	3.5	4.0	100.0
	FLC	0.5	50	
*C. pelliculosa*	*Ca*Thi	15.0	2.63	57.45
	FLC	2.5	7.8	
*C. buinensis*	*Ca*Thi	5.0	19.0	67.01
	FLC	0.06	5.45	
*C. mogii*	*Ca*Thi	10.0	17.19	61.05
	FLC	1.0	22.80	

^a^ *Ca*Thi concentrations ranging 1.3 to 4.0 times below it IC_50_ and FLC concentrations 2.0 times below it IC_50_ or at it IC_50_

^b^Indicates significance by the ANOVA test (*P* < 0.05) which were calculated by the absorbance values of synergism among the experiments and their respective controls

### Morphological alterations of *Ca*Thi and FLC plus *Ca*Thi on yeast growth

**Fig. 5:**
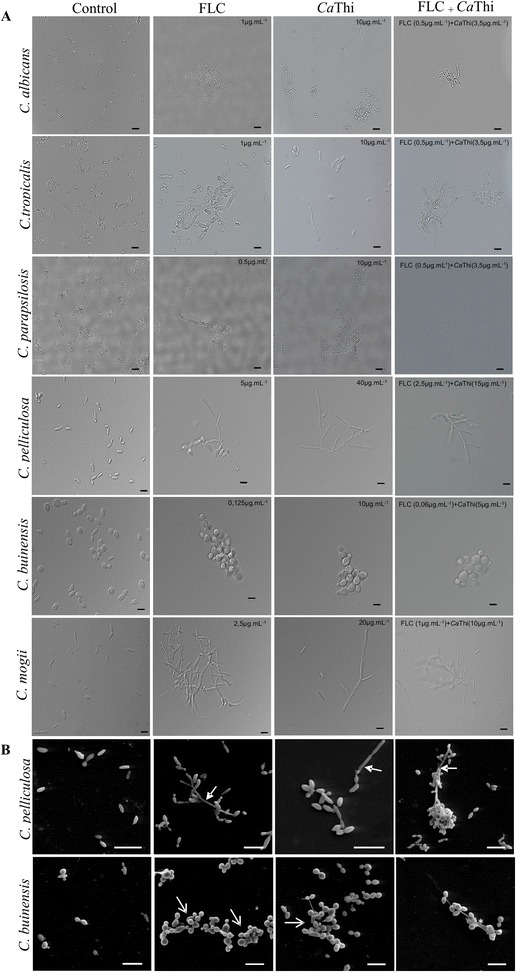
Effect of *Ca*Thi, FLC, and *Ca*Thi plus FLC on yeast growth. **a** Photomicrography of *Candida albicans*, *Candida tropicalis*, *Candida parapsilosis*, *Candida pelliculosa*, *Candida buinensis*, and *Candida mogii* cells by light microscopy after the growth inhibition assay. Bars 5 μm. **b** Scanning electron microscopy of *Candida pelliculosa* and *Candida buinensis*. Filled arrow (formation of pseudohyphae); open arrow (cell agglomeration); asterisk (amorphous material). Bars 10 μm. Cells grown in the presence of Fluconazole (FLC), *Ca*Thi, and FLC plus *Ca*Thi

Investigation regarding the possible morphological alterations in yeast cells grown in the presence of FLC, *Ca*Thi, or a combination of both substances after the inhibition assays (Fig. 5a) was performed. Optical microscopy analysis revealed that FLC, *Ca*Thi, and the combined treatment caused changes in the morphology of cells of *Candida* species. *C. tropicalis*, *C. parapsilosis*, *C. pelliculosa*, and *C. mogii* exhibited an apparent difficulty in releasing buds thus leading to the formation of pseudohyphae when grown in the presence of *Ca*Thi. Moreover, in the presence of either substance, *C. tropicalis* cells presented hyper branching of pseudohyphae. For *C. albicans* and *C. buinensis*, an intense cellular agglomeration in all treatments was observed. Further, the combination of *Ca*Thi and FLC caused a shrunken appearance in *C. albicans* cells.

Scanning Electronic Microscopy (SEM) of *C. pelliculosa* reinforces the optical microscopy observations, corresponding to intense cell agglomeration and pseudohyphae formation in all treatments. For *C. buinensis*, all treatments showed intense cellular agglomeration and apparent difficulty in bud release, but not in the formation of pseudohyphae. For this yeast an amorphous material among cells in all treatments was also observed (Fig. 5b). These results show that *Ca*Thi is capable of causing morphological changes similar to FLC, an azole antifungal agent, commonly used in treatment of infections caused by *Candida* species. Importantly, we were able to demonstrate that the combination of these substances potentiates the therapeutic effects against these opportunistic species of *Candida*.

## Discussion

Plant-derived thionins exhibit toxic effects against a wide range of plant pathogens including bacteria and fungi [18–21]. However, there is a gap regarding the mode of action of plant-derived thionins against human pathogens. Prompted by the considerable increase in the incidence of human infections by *Candida* species, we investigated the potential of *Ca*Thi, a plant-derived thionin peptide, as a novel therapeutic drug against six *Candida* strains of clinical interest: *C. albicans*, *C. tropicalis*, *C. parapsilosis*, *C. pelliculosa*, *C. buinensis*, and *C. mogii*.

The growth inhibition assay was not done with RPMI 1640 medium, which is generally indicated by Clinical and Laboratory Standards Institute guidelines, because it has in its composition a large amount of inorganic salts and it is well known in the literature that the presence of salts, such as sodium chloride and magnesium sulfate, can decrease the inhibitory activity of antimicrobial peptides since it is necessary electrostatic approximation of the peptide with the membrane of microorganisms and the presence of salts disrupts this initial interaction [22–24]. Therefore, as explained above, our growth inhibition assay was done with Sabouraud broth which is a common used medium to growth of fungi, including *Candida albicans*.

Our growth inhibition assays of the six *Candida* species tested revealed that 10 μg.mL^−1^ was IC_50_ for *Ca*Thi to inhibit the growth of *C. albicans*, *C. tropicalis*, *C. parapsilosis*, and *C. buinensis* but 40 μg.mL^−1^ was necessary to achieve IC_50_ for *C. pelliculosa* (Table 1), and this inhibitory effect was candidacidal inducing viability loss in all yeast cells tested (Fig. 1). Thi 2.1, a thionin from *Arabidopsis thaliana*, achieved 80 % inhibition of *C. albicans* with 2.5 μg.mL^−1^ [25]. Although Thi 2.1 showed stronger antimicrobial activity against *C. albicans* than *Ca*Thi, these authors did not test it against non-*albicans* species. Thus, whether this thionin would affect other species with similar strength remains unknown.

Sytox green is a dye that only penetrates cells when the plasma membrane is structurally compromised. All yeast species tested showed Sytox green fluorescence (Fig. 2), however *Ca*Thi was more effective in plasma membrane permeabilization in *C. albicans* and *C. pelliculosa* (Table 2). Antimicrobial activity against the fungus *Neurospora crassa* by α-Hodothionin, isolated from barley seeds, also occurs via a mechanism involving membrane permeabilization, resulting in the inward flux of Ca^2+^ and K^+^ efflux and consequent potential membrane collapse [26]. Another plant-derived thionin isolated from *Viscum album,* named VtA_3,_ interacts with the plasma membrane of the fungus *Fusarium solani*, causing its permeabilization and thus inhibiting the growth of this microorganism [27]. Indeed, several studies suggest that most of the biological effects of thionins result from the interaction of these peptides with the target cell membrane. Three mechanisms have been proposed: formation of an ion-selective channel; formation of patches or carpets of peptides; and loss of membrane phospholipids [10].

AMPs have been demonstrated to play a direct role in membrane permeabilization, causing a loss of membrane potential [28]. As cells depend on membrane potential to fulfill their physiological functions, its restoration is mandatory and demands cellular energy. One possible consequence of this process is ROS generation by activated mitochondria [29]. Therefore, we analyzed whether this primary membrane-permeabilizing event in *Candida* species induced by *Ca*Thi was followed by oxidative stress. Interestingly, *Ca*Thi only induced production of ROS in *C. tropicalis* (Fig. 3). We speculate that *Ca*Thi binds to a specific domain of the *C. tropicalis* membrane, which triggers the increase in oxidative stress response through ROS production. However, further studies are needed to establish this. Reports show that increase in ROS production by the target organisms is a recurring mode of action employed by thionins and other AMPs [27, 30, 31]. Indeed, increased death of the fungus *Fusarium solani* subjected to VtA_3_ was connected with boosted ROS production by these organisms [27]. More recently, a peptide similar to thionin isolated from the beetle *Psacothea hilaris* also provoked in increase in the levels of endogenous ROS in *C. albicans* [32]. Our study is the first to report increase in ROS production by a plant-derived thionin as an antimicrobial mechanism against a human pathogen, *C. tropicalis*. Therefore, *Ca*Thi seems to employ a sophisticated mechanism to inhibit the growth of this opportunistic yeast involving not only membrane permeabilization but also induction of oxidative stress response.

Some AMPs are able to enter the cell, after the initial cell membrane interaction [33, 34]. Accordingly, the next experiments were designed to analyze whether *Ca*Thi was able to actively enter *C. albicans* and *C. tropicalis*. In the approach used, FITC-tagged *Ca*Thi was monitored by fluorescence microscopy. Because *Ca*Thi entered *C. albicans* and *C. tropicalis* cells, we suggest that a possible intracellular target for this thionin might be part of a complex mechanism responsible for the death of *Candida* species. FITC-tagged *Ca*Thi overlapped with DAPI staining, indicating that, in *C. tropicalis*, this target is nuclear (Fig. 4). Giudici et al. [27] showed that VtA_3_ entered and accumulated in the fungus *F. solani*. These authors also demonstrated that this entry was related to the sphingolipid composition of the plasma membrane of this fungus. Our study is the first to show that a plant-derived thionin (*Ca*Thi) is able to enter human pathogens (*C. albicans* and *C. tropicalis*) and to suggest an intracellular target for it. Our work opens new perspectives regarding the antimicrobial mechanism of plant-derived thionins as it suggests that these peptides’ toxicity may not be restricted to the plasma membrane.

The real mode of action of AMPs has not been fully elucidated, but much of the described AMPs to date target the plasma membrane of microorganisms causing pore formation and leading to imbalance in cellular homeostasis [33, 35]. However, some studies showed that not only is permeabilization the cause of a particular microorganism death, as they may have multiple targets [36] after the interaction with the membrane causing, for example, ROS induction [31, 37]; inhibition of protein synthesis [38, 39]; inhibition of mitochondrial activity [40, 41], and also may trigger signaling cascades that lead to apoptosis [42, 43]. Thus it is difficult to identify the most important factor for the candidacidal effect of *Ca*Thi and is technically challenging to characterize the steps leading up to cell death, however evidence supports that all events described in the manuscript may have a crucial role in the death of the tested yeasts.

The continuous emergence of resistance of fungal strains to conventional antibiotics and antifungals, especially *Candida* species, has become an important medical issue and has spurred the demand for new therapeutic alternatives. This concern prompted us to investigate whether FLC and *Ca*Thi could act synergistically to improve therapeutic results against *Candida* species. Here we show that the combination of these two substances was effective against all *Candida* species tested (Table 3), causing drastic morphological changes in these cells (Fig. 5). Interestingly, we show that the inhibitory effect of this combination was more effective for *C. albicans*, *C. parapsilosis*, and *C. tropicalis*, which are the major yeast species recovered from infections in immunocompromised patients [13]. The azoles mode of action occurs by inhibition of the enzyme lanosterol 14 α-demethylase, blocking ergosterol incorporation and leading to the accumulation of intermediate sterols. These intermediate sterols do not have the same configuration and physical properties of ergosterol, therefore they cause the plasma membrane to form with altered properties, changing in fluidity, permeability and impairing nutrient uptake, which ultimately lead to cell toxicity [44, 45]. In regard to the synergistic effect of FLC and *Ca*Thi, we hypothesize that permeabilization is firstly caused by *Ca*Thi (Fig. 2) facilitating the entrance of FLC into the cell cytoplasm, triggering structural alterations in the plasma membrane which feedback positively to the entrance of more *Ca*Thi and FLC. This entrance creates potential for toxic effects, which were experimentally observed in the lower IC_50_ used for both substances in the combinatory treatment (Table 3). Additionally, secondary toxicity effects were demonstrated, such as the induction of ROS to *C. tropicalis,* the *Ca*Thi presence in *C. albicans* cell cytoplasm and in *C. tropicalis* nuclei. These localizations suggest that *Ca*Thi may have cytoplasmic targets, where interference could consolidate the inhibitory effect. However, more studies are necessary to clearly unravel the antimicrobial mechanism of *Ca*Thi against *Candida* species as well as the mechanism of synergy between *Ca*Thi and FLC.

## Conclusions

Investigating a plant-derived thionin mode of action against opportunistic human pathogenic yeasts is relevant and advisable, whereas most studies involving plant-derived thionins focus their effects against plant pathogenic microorganisms as experimental models. In this report, we demonstrated that *Ca*Thi has strong candidacidal activity against six pathogenic *Candida* species, and it works by permeabilizing the membrane and inducing oxidative stress response in these yeasts. Additionally, we present evidence to suggest a nuclear intracellular target for *Ca*Thi. Finally, our results show that FLC and *Ca*Thi combined causes dramatic morphological changes in these yeasts, effective against all *Candida* species tested. The combined treatment of *Ca*Thi and FLC is a strong candidate for clinical studies aiming to improve therapeutic results against resistant strains of *Candida* species. Studies involving drug combinations should be reinforced due to the possibility of synergistic effects that increase the toxic effect of the drugs combined when compared to monotherapy. Moreover, drug combinations can broaden the spectrum of antimicrobial activity, minimizing resistant microorganisms selection, increasing security and tolerance using lower drugs doses.

## Methods

### Biological materials

*Capsicum annuum* L. fruits (accession UENF1381) were provided by *Laboratório de Melhoramento Genético Vegetal*, from *Centro de Ciências e Tecnologias Agropecuárias*, *Universidade Estadual do Norte Fluminense Darcy Ribeiro* (UENF), Campos dos Goytacazes, Rio de Janeiro, Brazil.

The yeasts *Candida albicans* (CE022), *Candida tropicalis* (CE017), and *Candida parapsilosis* (CE002) were obtained from *Departamento de Biologia*, *Universidade Federal do Ceará*, Fortaleza, Brazil. The yeasts *Candida pelliculosa* (3974), *Candida buinensis* (3982), and *Candida mogii* (4674) were obtained from *Micoteca URM* from *Universidade Federal de Pernambuco*, Recife, Pernambuco, Brazil. Yeasts were maintained on Sabouraud agar (1 % peptone, 2 % glucose, and 1.7 % agar-agar) (Merck) in the *Laboratório de Fisiologia e Bioquímica de Microrganismos*, from *Centro de Biociência e Biotecnologia*, UENF, Campos dos Goytacazes, Rio de Janeiro, Brazil.

### *Ca*Thi

Extraction and purification of the thionin *Ca*Thi from *Capsicum annuum* fruits by chromatographic methods were performed as described by Taveira et al. [8]. The retention time to recover the thionin *Ca*Thi during the reversed-phase chromatography in column μRPC C2/C18 (ST 4.6/100) (GE Healthcare) was 37.87 min.

### Preparation of yeast cells and determination of IC_50_ for *Ca*Thi and fluconazole

For the preparation of yeast cell cultures, an inoculum from each stock of *Candida albicans*, *Candida tropicalis*, *Candida parapsilosis, Candida pelliculosa*, *Candida buinensis*, and *Candida mogii* was transferred to Petri dishes containing Sabouraud agar and allowed to grow at 30 °C for 48 h. After this time, each cell aliquot was added to 10 mL sterile culture medium (Sabouraud broth, Merck). The cells were quantified in a Neubauer chamber (Optik Labor) with the aid of an optical microscope (Axiovison 4, Zeiss). The assay for checking the growth inhibition of yeast cells was performed according to Broekaert et al. [46] with modifications. Initially yeast cells (1 x 10^4^ cells mL^−1^) were incubated in Sabouraud broth containing *Ca*Thi at concentrations ranging from 100 μg.mL^−1^ to 1 μg.mL^−1^ and fluconazole (FLC) at concentrations ranging from 20 μg.ml^−1^ to 0.125 μg.mL^−1^, with the final volume adjusted to 200 μL. The assay was performed in 96-well microplates (Nunc) at 30 °C for 24 h. Optical readings at 620 nm were recorded at zero hour and every 6 h interval for 24 h. Control cells were grown in the absence of *Ca*Thi and FLC. The optical densities were plotted against the concentration of *Ca*Thi and FLC, and then the concentration of the drug (*Ca*Thi and FLC) required for 50 % inhibition (IC_50_) *in vitro* of the tested yeasts was determined. Experiments were performed in triplicate.

### Viability assay

To assay the effect of *Ca*Thi on the cell viability of yeasts, 1x10^4^ cells mL^−1^ in Sabouraud broth culture medium and at the corresponding IC_50_ of *Ca*Thi values for each yeast were incubated at 30 °C for 24 h in 96-well microplates (Nunc). To determine the control viability, the control cells (without *Ca*Thi) were washed once and diluted 1,000–fold in Sabouraud broth culture medium, and an aliquot of 100 μL from this dilution was spread over the surface of a Sabouraud agar medium (contained in a Petri dish) with a Drigalski loop and grown at 30 °C for 48 h. At the end of this period, colonies forming units (CFU) were determined for each yeast species, and the Petri dishes were photographed. The same procedure was followed with yeasts treated with *Ca*Thi. The experiments were carried out in triplicate, and the results are shown assuming that the control represents 100 % viability. Calculations of the standard deviation and *T* test were performed with Prism software (version 5.0).

### Plasma membrane permeabilization assay

The plasma membrane permeabilization of yeast cells was measured by Sytox green uptake, according to the methodology described by Thevissen et al. [47], with some modifications. Each of the different species of yeasts was incubated with *Ca*Thi at the concentration required to inhibit 50 % growth (IC_50_) of the respective yeast cells for 24 h. After this time, a 100 μL aliquot of each yeast cell suspension was incubated with 0.2 μM of Sytox green in 1.5 mL microcentrifuge tubes for 30 min at 25 °C with constant agitation. Cells were analyzed by DIC optical microscope (Axiovison 4, Zeiss) equipped with a fluorescent filter set for detection of the fluorescein (excitation wavelength, 450–490 nm, emission wavelength 500 nm). To indicate membrane permeabilization, the percentage of fluorescent cells was determined by counting the DIC and fluorescent views for each yeast (*n* = 5). The total cell number in DIC view of each yeast (in control and test) was assumed as 100 %. The experiments were carried out in triplicate. Calculations of *T* test were performed with Prism software (version 5.0).

### Determining the induction of intracellular ROS in yeast cells

To evaluate whether the mechanism of action of *Ca*Thi involves induction of oxidative stress, the fluorescent probe 2′, 7′-dichlorofluorescein diacetate (H_2_DCFDA) was used to measure intracellular reactive oxygen species (ROS) according to the methodology described by Mello et al. [31]. Each of the different species of yeasts was incubated with the respective IC_50_ for *Ca*Thi in 96-well microplates for 24 h at 30 °C; after this incubation an aliquot of 50 μL of each of yeast cell suspension was incubated with 200 μM of H_2_DCFDA in micro centrifuge tubes of 1.5 mL for 1 h at 25 °C with constant agitation at 500 rpm. Cells were analyzed by DIC optical microscope (Axiovison 4, Zeiss) equipped with a fluorescent filter set for detection of the fluorescein (excitation wavelength, 450–490 nm, emission wavelength 500 nm). The experiments were carried out in triplicate.

### *Ca*Thi conjugated to FITC localization for optical microscopy

*Ca*Thi at 100 μg was resuspended in 100 μL of 750 mM sodium carbonate-sodium bicarbonate buffer, pH 9.5 containing FITC at 50 μg.mL^−1^ (previously solubilized in DMSO). This solution was incubated with constant agitation at 500 rpm for 2 h at 30 °C. After this incubation, the sample was submitted to gel filtration chromatography on Sephadex G25 column (Sigma) for elimination of free FITC and recovery *Ca*Thi-FITC. The column was equilibrated and run with 20 mM Tris–HCl, pH 8.0 at flow rate of 0.3 mL.mim^−1^. After coupling, 10 μg.mL^−1^ of *Ca*Thi-FITC was incubated with cells of *C. albicans* and *C. tropicalis* for 24 h in 96-well microplates. After this time an aliquot of each cell suspension was removed and incubated with 50 μg.mL^−1^ of 4′,6-diamidino-2-phenylindole dihydrochloride (DAPI) for 10 min for nuclei stain. Cells were analyzed by DIC optical microscope (Axiovison 4, Zeiss) equipped with a fluorescent filter set for detection of the fluorescein (excitation wavelength, 450–490 nm, emission wavelength 500 nm). The entire assay was performed protected from light.

### Synergism assay

To verify the synergistic activities, we combined FLC with *Ca*Thi. Initially yeast cells (1 x 10^4^ cells mL^−1^) were incubated in Sabouraud broth containing an IC_50_ concentration or less than that for FLC and *Ca*Thi concentrations ranging from 1.3 to 4.0 times below the IC_50_ for the respective yeast and the final volume adjusted to 200 μL *in vitro*. The assay was performed in 96-well microplates (Nunc) at 30 °C for 24 h. Optical readings at 620 nm were taken at zero hour and every 6 h for the following 24 h. Control cells were: 1) grown in the absence of *Ca*Thi and FLC; 2) grown in the presence of FLC; 3) grown in the presence of *Ca*Thi. The synergistic activity was deduced comparing optical densities of each control and combined drugs (FLC plus *Ca*Thi) considering each yeast strain tested. We define synergism as the combination action of the AMP with other substance that causes an enhanced decrease in the growth of the microorganism, compared with the growth inhibition of the single substances. After synergism, assay cells (controls and tests) were analyzed by DIC optical microscope (Axiovison 4, Zeiss). The data were obtained from experiments performed in triplicate. The data were evaluated using a one-way ANOVA. Mean differences at *p* < 0.05 were considered to be significant. All statistical analyses were performed using the GraphPad Prism software (version 5.0 for Windows).

### Scanning electron microscopy

*C. pelliculosa* and *C. buinensis* cells were submitted to scanning electron microscopy (SEM) analysis. Yeast cells were grown for 24 h in Sabouraud broth in the presence of FLC (5 μg.mL^−1^ and 0.125 μg.mL^−1^, respectively), *Ca*Thi (40 μg.mL^−1^ and 10 μg.mL^−1^, respectively), and FLC plus *Ca*Thi (2.5 μg.mL^−1^ + 15 μg.mL^−1^, 0.06 μg.mL^−1^ + 5 μg.mL^−1^, respectively) or absence of these drugs, then were fixed for 30 min at 30 °C in a solution containing 2.5 % glutaraldehyde and 4.0 % formaldehyde in 0.1 M cacodylate buffer, pH 7.0. Subsequently, the cells were rinsed three times in 0.1 M cacodylate buffer, pH 7.0; post-fixed for 30 min at 30 °C with 1.0 % osmium tetroxide diluted in 0.1 M cacodylate buffer, pH 7.0; and rinsed again with this same buffer. The yeast cells were gradually dehydrated in alcohol solution (15, 30, 50, 70 to 90 % and finally 100 % alcohol), critical point dried in CO_2_, covered with 20 nm gold and observed in a DSEM 962 Zeiss SEM.

## Abbreviations

AMP: Antimicrobial peptide
FLC: Fluconazole
ROS: Reactive oxygen species
*Ca*Thi: *Capsicum annuum* Thionin
DIC: Differential interference contrast
FITC: Fluorescein isothiocyanate
SEM: Scanning Electron Microscopy
